# Targeting the microenvironment in the treatment of arteriovenous malformations

**DOI:** 10.1007/s10456-023-09896-3

**Published:** 2023-09-21

**Authors:** Caroline T. Seebauer, Benedikt Wiens, Constantin A. Hintschich, Natascha Platz Batista da Silva, Katja Evert, Frank Haubner, Friedrich G. Kapp, Christina Wendl, Kathrin Renner, Christopher Bohr, Thomas Kühnel, Veronika Vielsmeier

**Affiliations:** 1 Department of Otorhinolaryngology, University Hospital Regensburg, Franz-Josef-Strauß-Allee 11, 93053 Regensburg, Germany; 2 Institute of Radiology, University Hospital Regensburg, Franz-Josef-Strauß-Allee 11, 93053 Regensburg, Germany; 3 Institute of Pathology, University of Regensburg, Franz-Josef-Strauß-Allee 11, 93053 Regensburg, Germany; 4 Department of Otorhinolaryngology, Ludwig Maximilian University of Munich, Marchioninistr. 15, 81377 Munich, Germany; 5 Division of Pediatric Hematology and Oncology, Department of Pediatrics and Adolescent Medicine, Medical Center, University of Freiburg, Heiliggeiststr. 1, 79106 Freiburg im Breisgau, Germany

**Keywords:** Arteriovenous malformations, Fast Flow malformations, Bevacizumab, Thalidomide, VEGF, VEGF inhibitor, Mechanical stress, Microenvironment

## Abstract

Extracranial arteriovenous malformations (AVMs) are regarded as rare diseases and are prone to complications such as pain, bleeding, relentless growth, and high volume of shunted blood. Due to the high vascular pressure endothelial cells of AVMs are exposed to mechanical stress. To control symptoms and lesion growth pharmacological treatment strategies are urgently needed in addition to surgery and interventional radiology. AVM cells were isolated from three patients and exposed to cyclic mechanical stretching for 24 h. Thalidomide and bevacizumab, both VEGF inhibitors, were tested for their ability to prevent the formation of circular networks and proliferation of CD31^+^ endothelial AVM cells. Furthermore, the effect of thalidomide and bevacizumab on stretched endothelial AVM cells was evaluated. In response to mechanical stress, VEGF gene and protein expression increased in patient AVM endothelial cells. Thalidomide and bevacizumab reduced endothelial AVM cell proliferation. Bevacizumab inhibited circular network formation of endothelial AVM cells and lowered VEGF gene and protein expression, even though the cells were exposed to mechanical stress. With promising in vitro results, bevacizumab was used to treat three patients with unresectable AVMs or to prevent regrowth after incomplete resection. Bevacizumab controlled bleeding, pulsation, and pain over the follow up of eight months with no patient-reported side effects. Overall, mechanical stress increases VEGF expression in the microenvironment of AVM cells. The monoclonal VEGF antibody bevacizumab alleviates this effect, prevents circular network formation and proliferation of AVM endothelial cells in vitro. The clinical application of bevacizumab in AVM treatment demonstrates effective symptom control with no side effects.

**Graphical abstract:**

Mechanical stress increases VEGF expression in endothelial AVM cells, possibly causing the VEGF upregulation in the microenvironment of AVM cells. The resulting RAS/RAF/MEK/ERK signaling in leads to progression of fast-flow malformations. The monoclonal VEGF-A antibody bevacizumab alleviates this effect, prevents circular network formation and proliferation of AVM endothelial cells in vitro. Sporadically occurring slow-flow malformations (LMs, VMs) have mutations in *TEK* or *PIK3CA*. *TEK* encodes the endothelial receptor tyrosine kinase TIE2. Sporadic extracranial fast-flow malformations (AVMs) show mutations in *KRAS, BRAF* and *MAP2K1*, which encodes the dual specificity mitogen-activated protein kinase MEK1. Combined targeting of the molecular causes of the disease could be key to achieve symptom control and reduce lesion growth. Orange: gain-of-function; Blue, circled with orange: enhanced signaling.

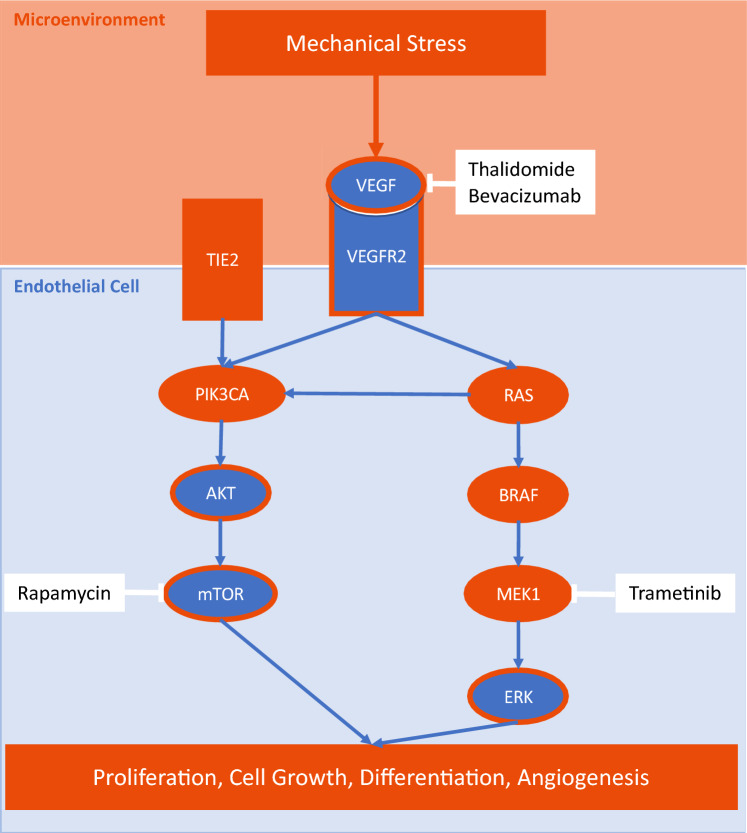

**Supplementary Information:**

The online version contains supplementary material available at 10.1007/s10456-023-09896-3.

## Introduction

Fast-flow extracranial arteriovenous malformations (AVMs) are characterized by direct connections between arteries and veins without an intervening capillary bed. These tangles of abnormal blood vessels are called nidus. The pathophysiology results in high-pressure blood flow through the AVM nidus, which can lead to a variety of symptoms, including pain, bleeding, relentless growth, cosmetic disfigurement, and high volume of shunted blood [1]. Due to high vascular pressure and blood flow, endothelial cells are exposed to mechanical stress. The effect of mechanical stress on AVMs is not fully understood, but it is believed to play a role in their development, progression, and potential complications [2]. In contrast, slow-flow malformations are characterized by abnormal veins, capillaries, or lymphatics with lower flow rates and less turbulent flow compared to AVMs, which results in less hemodynamic stress on the vascular walls. This aligns with a more stable growth pattern observed in slow malformations [3, 4].

Treatment for extracranial AVMs involves surgery and embolization. While these interventions are effective in reducing the size and symptoms of the AVM, recurrence rates can be significant [5]. Factors such as incomplete removal of the AVM, residual blood flow, and the development of new feeder vessels contribute to recurrence [6].

In recent years, there has been growing interest in the use of antiangiogenic drugs, such as thalidomide and bevacizumab, to treat AVMs [7]. Bevacizumab is a monoclonal antibody that binds to vascular endothelial growth factor (VEGF), a key mediator of angiogenesis [8, 9]. Thalidomide has been shown to inhibit angiogenesis by targeting basic fibroblast growth factor (bFGF), transforming growth factor-beta (TGF-β) and VEGF [10]. Due to its cytokine inhibition it prevents capillary microvessel formation, endothelial cell migration, and increases cell adhesion to collagen [11].

To control symptoms, growth of unresectable AVMs, and recurrence after resection or embolization targeted therapies are needed. Therefore, we investigated how mechanical stress affects endothelial cells of AVMs and their microenvironment. Further, we tested if thalidomide and bevacizumab, as antiangiogenic drugs, can mitigate this effect.

## Results

### Upregulation of VEGF and VEGF receptors in fast-flow malformations

**Fig. 1:**
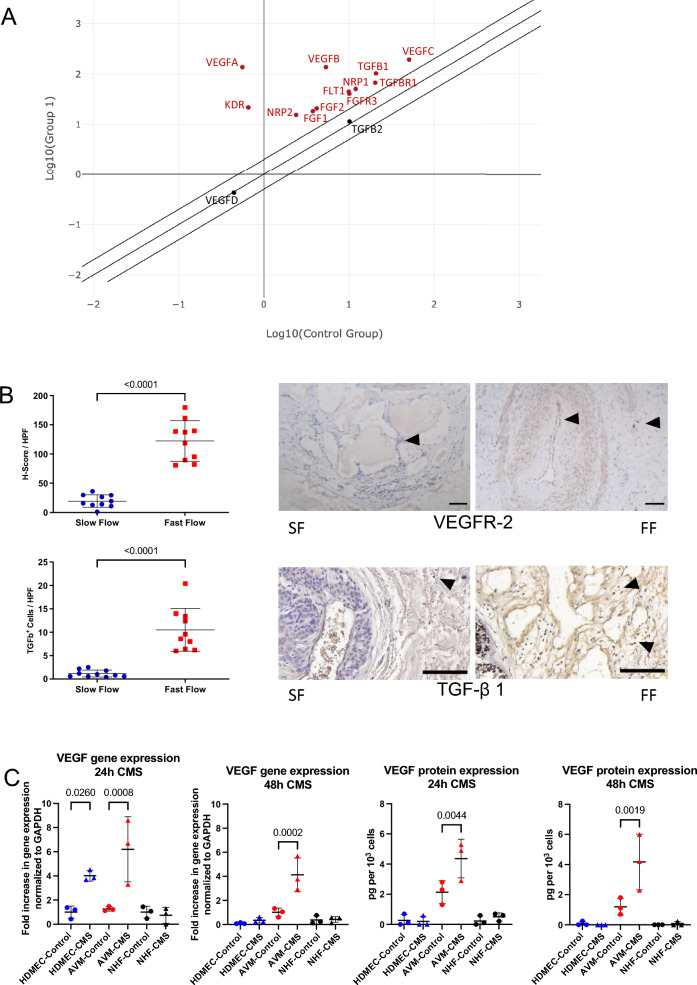
Upregulation of VEGF and VEGFR in fast-flow malformations. **A** PCR array analysis of angiogenesis-associated genes of fast-flow (n = 10; Group1) and slow-flow (n = 10; Control Group) patients shows an upregulation of VEGF (VEGFA, VEGFB, VEGFC) and VEGF-receptors (VEGFR-1 = FLT1, VEGFR-2 = KDR, VEGF co-receptors NRP-1 and NRP-2), as well as an upregulation of angiogenic signaling molecules FGF-1, FGF-2, TGF-β 1 and their receptors (TGF-β receptor 1 and FGFR 3) in fast-flow malformations. Scatter plot shows fold change, which is the normalized (2 − ΔCt) gene expression in fast-flow lesions (Group 1) divided by the normalized gene expression in slow-flow lesions (Group 2). Upregulated genes are highlighted red. Beta-actin (ACTB) functions as housekeeping gene. **B** Immunohistochemical staining of fast-flow (n(AVM) = 10) and slow-flow (n(VM) = 6; n(LM) = 4) malformations reveals an upregulation of VEGFR-2 and TGF-β 1 in the microenvironment of fast-flow malformations. The H-score assesses the extent of immunoreactivity of the VEGF receptor staining. P-values displayed were calculated by Mann Whitney test. Means and standard deviations are shown. HPF = high-power fields; SF = slow flow malformation; FF = fast flow malformation. Scale bar, 100 μm **C** Exposed to cyclic mechanical stretching (CMS), AVM CD31^+^ endothelial cells show an upregulation of VEGF on mRNA and protein levels after 24 h and 48 h (for all p-values see Supplemental Table 1C). Human endothelial cells (HDMEC) and fibroblasts (NHF) do not respond to mechanical stress after 48 h. P-values displayed were calculated by one-way ANOVA followed by the post hoc Šidák test for multiple comparisons. Means and standard deviations are shown

We hypothesized, that the hemodynamic stress on the vascular walls of fast-flow malformations (FF) induces a pattern of cytokines that drives angiogenesis and induces the lesion’s unique growth behavior. Therefore, a polymerase chain reaction (PCR) array of angiogenesis-associated genes was performed (Fig. 1A; for summary of all PCR array results see Supporting Fig. 1A). Comparing multiple samples of ten pooled fast-flow malformation patients (arteriovenous malformations = “Group 1”) to ten pooled slow-flow malformation (SF) patients (venous and lymphatic malformations = “Control Group”) an upregulation of VEGFA, VEGFB, VEGFC, VEGF-receptor 1 (VEGFR-1 = FLT1), VEGF-receptor 2 (VEGFR-2 = KDR) and the VEGF co-receptors Neuropilin-1 (NRP-1) and Neuropilin-2 (NRP-2) was detected in fast-flow malformations. Furthermore, the level of the angiogenic signaling molecules FGF-1, FGF-2, TGF-β 1, and their receptors TGF-β receptor 1 and FGF receptor 3 were elevated as well (Fig. 1A). Immunohistochemistry was performed to detect the expression of VEGFR-2 and TGF-β in the microenvironment of fast-flow (arteriovenous malformations (n = 10) and slow-flow malformations (venous (n = 6) and lymphatic malformations (n = 4)). Quantification revealed a significant overexpression of VEGFR-2 and TGF-β 1 in the microenvironment surrounding fast-flow lesions compared to slow-flow malformations (Fig. 1B). To determine, if the detected VEGF expression in fast-flow malformations is caused by hemodynamic stress, cells of three AVM lesions were isolated and exposed to cyclic mechanical stretching (CMS) for 24 or 48 h. The AVM endothelial cells were selected using anti-CD31-coated magnetic beads. In all three AVM patients *KRAS* mosaic mutations were detected. Human dermal endothelial cells (HDMEC) and normal human fibroblasts (NHF) were exposed to cyclic mechanical stretching as controls. A three-fold increase of VEGF was detected in HDMEC within 24 h on mRNA levels but did not translate into elevated protein levels. Mechanical stress did not affect HDMEC and NHF after 48 h. Exposure to cyclic mechanical stretching induced a significant mRNA and protein expression of VEGF only in endothelial AVM cells (Fig. 1C; for p values see Supplemental Table 1C).

### Bevacizumab and thalidomide mitigate VEGF induced effects in endothelial AVM cells

**Fig. 2:**
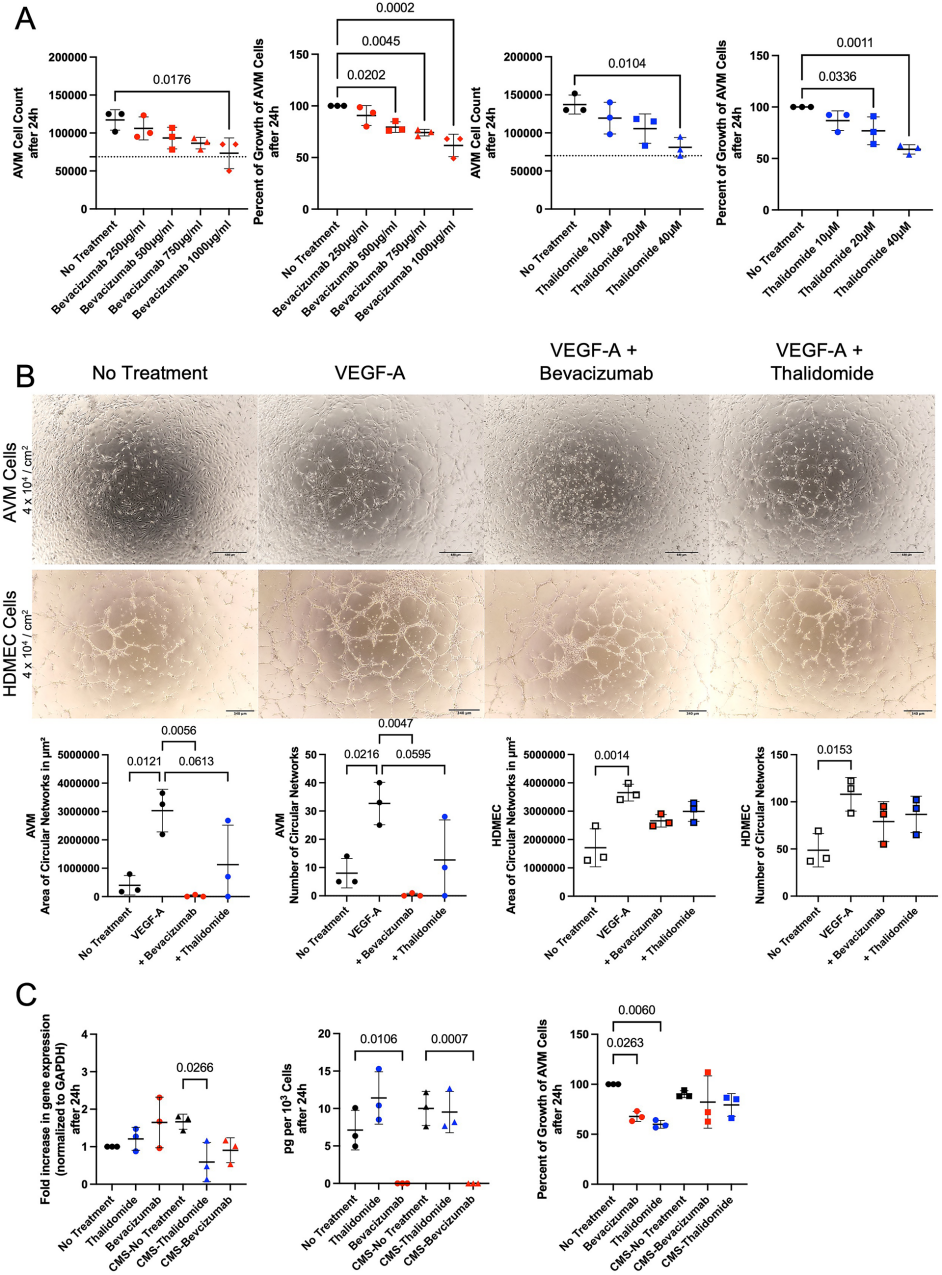
Bevacizumab and thalidomide alleviate VEGF induced effects in AVM endothelial cells. **A** Ascending concentrations of bevacizumab and thalidomide significantly inhibit proliferation of CD31^+^ endothelial AVM cells after 24 h of treatment. Dashed line indicates 4-h attachment level (mean) of AVM endothelial cells. **B** Treatment of CD31^+^ endothelial AVM cells with bevacizumab (1000 µg/ml) inhibits VEGF-A (25 ng/ml) induced circular network formation of AVM endothelial cells. Thalidomide (20 µM) treatment does not reduce circular network formation to an equal extent. Scale bar for AVM pictures, 680 μm. Scale bar for HDMEC pictures, 340 μm. **C** Bevacizumab (1000 µg/ml) and thalidomide (20 µM) inhibit VEGF overexpression in CD31^+^ endothelial AVM cells in response to cyclic mechanical stretching (CMS) (first panel). VEGF protein levels are reduced by bevacizumab (1000 µg/ml), not by thalidomide (20 µM) (middle panel). Bevacizumab (1000 µg/ml) or thalidomide (20 µM) treatment does not reduce cell proliferation of AVM endothelial cells exposed to mechanical stress (last panel). P-values displayed were calculated by one-way ANOVA followed by the post hoc Bonferroni test for multiple comparisons (for all p-values see Supplemental Table 2A-C). Means and standard deviations are shown

Our results demonstrated that mechanical stress led to an upregulation of VEGF and TGF-β in the microenvironment of AVMs. Therefore, we hypothesized, that antiangiogenic drugs like the monoclonal IgG1 antibody against VEGF-A bevacizumab and the growth factor targeting inhibitor thalidomide alleviate this effect. To determine, how bevacizumab and thalidomide affect endothelial AVM cell proliferation, endothelial AVM cells were treated with both drugs for 24 h in multiple drug concentrations. Concentrations were tested in accordance with current literature [12–15]. Neither bevacizumab, nor thalidomide, altered endothelial AVM cell attachment 4 h after cell seeding (Supporting Fig. 2 A). Untreated endothelial AVM cells underwent roughly one population doubling within 20 h (1.89-fold increase of attached cells, determined after 4 h and 24 h). The proliferation assay revealed a significant inhibition of endothelial AVM cell proliferation with 1000 µg/ml bevacizumab (37.4% reduction of growth compared to untreated cells) and 20 µM thalidomide treatment (23.0% reduction of growth compared to untreated cells) after 24 h (Fig. 2A; for p values see Supplemental Table 2A). This effect is VEGF specific, as treatment of endothelial AVM cells with a monoclonal IgG1 antibody against tyrosine-protein kinase Met (onartuzumab) did not decrease cell proliferation (Supporting Fig. 2B).

An angiogenesis assay demonstrated that 1000 µg/ml bevacizumab inhibited the formation of circular networks by endothelial AVM cells (98.98% reduction of the number of circular networks and 99.26% reduction of area of circular networks compared to untreated cells). 20 µM thalidomide did not show an equally strong inhibition neither of number nor of area of circular networks in this assay (61.22% reduction of number of circular networks and 62,80% reduction of area of circular networks compared to untreated cells) (Fig. 2B upper panel). Moreover, only two of the patient samples showed a decrease in circular network formation, whereas one sample of an AVM patient did not respond with reduced circular network formation to thalidomide treatment. Therefore, no significant reduction was observed in this treatment group. HDMECs served as positive control (Fig. 2B middle panel; for p values see Supplemental Table 2B). Bevacizumab and thalidomide did not impair the ability of HDMECs to form circular networks when exposed to VEGF-A.

After assessing the effect of bevacizumab and thalidomide on endothelial AVM cells by functional assays, we performed biochemical assays to understand the mechanism of action. Therefore, we exposed CD31^+^ endothelial cells of three AVM lesions to mechanical stress and evaluated whether 1000 µg/ml bevacizumab and 20 µM thalidomide prevented increased VEGF expression during a 24-h period, as described above. VEGF m-RNA and protein levels were measured by qPCR and ELISA, respectively. The mechanical stress induced VEGF overexpression in endothelial AVM cells was inhibited by bevacizumab (45.6% reduction compared to untreated cells) and thalidomide (64.6% reduction compared to untreated cells) on mRNA level (Fig. 2C, first panel). A significant reduction of the VEGF protein levels was only achieved by bevacizumab, but not thalidomide (Fig. 2C, middle panel). Cell proliferation of endothelial AVM cells exposed to cyclic mechanical stretching was not reduced by bevacizumab or thalidomide treatment. Bevacizumab (32.2% reduction compared to untreated cells) and thalidomide (40.1% reduction compared to untreated cells) only inhibited cell proliferation of endothelial AVM cells not exposed to mechanical stress (Fig. 2C, last panel; for p values see Supplemental Table 2C).

### Bevacizumab treatment results in effective symptom control in three AVM patients

From our in vitro results we concluded that the mechanical stress of the high pressure on the vascular walls of AVMs induces a VEGF upregulation in the microenvironment of the lesion, which might contribute to disease progression. Bevacizumab reduced the increased VEGF protein levels expressed by AVM endothelial cells in vitro. Therefore, after interdisciplinary and multicentric discussion we decided to treat three patients with bevacizumab. Other therapeutic options, like embolization and surgery, did not result in symptom control in these patients.

**Fig. 3:**
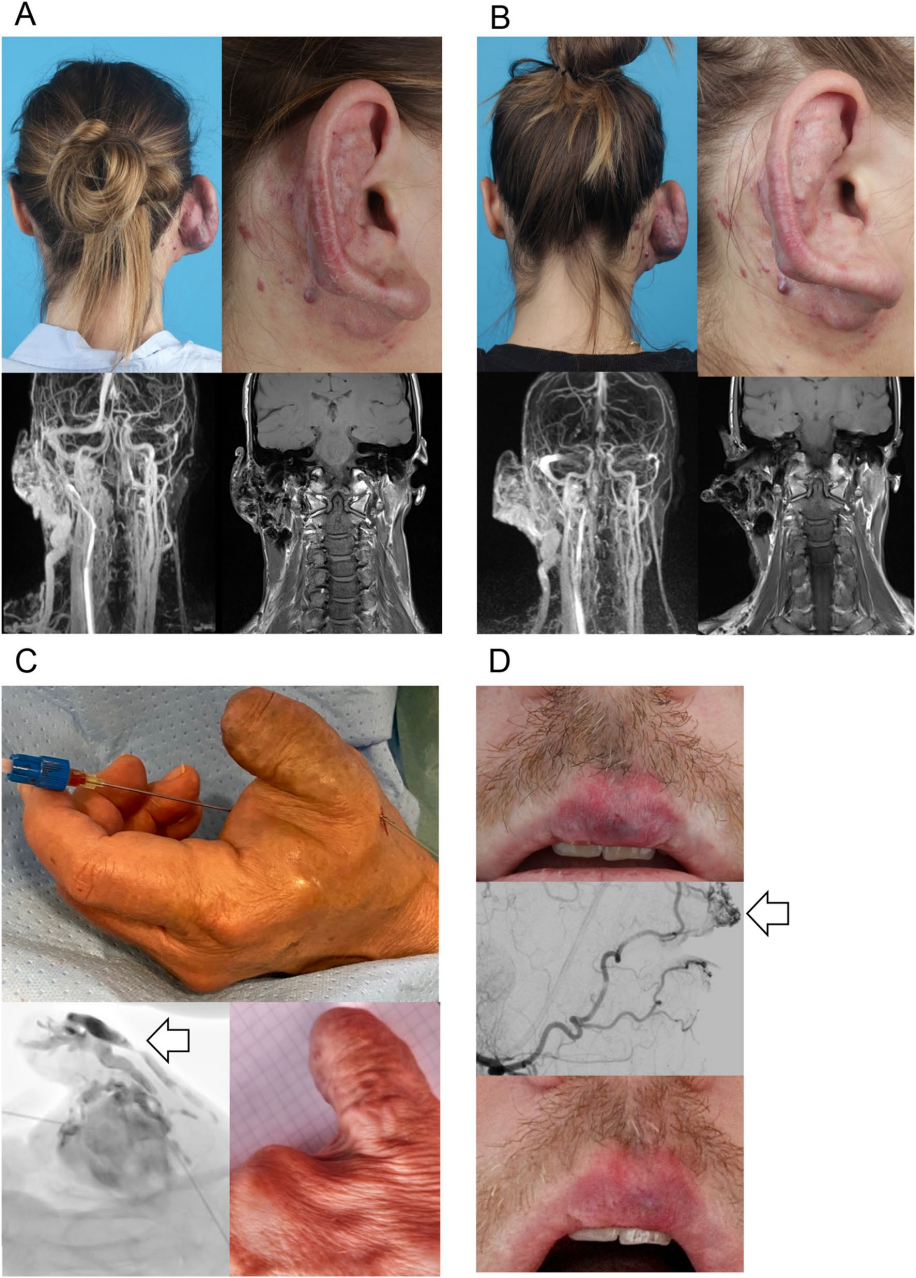
Bevacizumab in AVM patient treatment results in effective symptom control **A** 25-year-old female patient with an AVM of the right ear and face (upper panel). MR angiography with contrast enhancement showing feeders from the right external occipital artery and posterior auricular artery with drainage to the external jugular vein (lower panel). **B** Clinical follow-up after eight months of bevacizumab treatment (5 mg/kg/bodyweight systemically every 14 days) demonstrated a brightening of lesion with reduction of pulsation and cessation of bleeding and pain (upper panel). The follow-up contrast enhanced MR angiography described minimal lesion growth (lower panel). **C** 66-year-old female patient with local bevacizumab injection into an AVM of the left hand (upper panel) to prevent regrowth of the lesion. DSA showing an AVM of the left thumb (white arrow) with feeders from the left radial artery drained by the left V. radialis et ulnaris (lower left panel). Five months after treatment the lesion is stable in size without bleeding, pulsation, or pain (lower right panel). **D** 35-year-old male patient with local bevacizumab injection into an AVM of the upper lip for symptom control (upper panel). DSA showing an AVM of the middle upper lip (white arrow) with feeders from the lingual and maxillary artery drained by the labial vein (sagittal view, middle panel). Three months after local bevacizumab a brightening and volume reduction of the lesion is visible (lower panel)

A 25-year-old female patient with an AVM of the right ear and face presented at the hospital with severe bleeding, pulsation, itching and pain from the lesion (Fig. 3A upper panel). She had previous treatment with Onyx embolization four years ago, which could not alleviate her symptoms. Prior to treatment initiation, a contrast enhanced MR angiography was performed, showing feeders from the right external occipital artery and posterior auricular artery with drainage to the external jugular vein (Fig. 3A lower panel). A biopsy from the lesion was taken to confirm the diagnosis and to perform genetic testing. To bridge the time until availability of the genetic test results the patient was treated with 5 mg bevacizumab per kg/bodyweight systemically every 14 days (395 mg bevacizumab intravenously every 14 days). The dosage was chosen as previously described for the treatment of gastrointestinal telangiectasias in hereditary hemorrhagic telangiectasia (HHT) [16]. The patient received treatment with bevacizumab for eight months. She reported dry skin and intermenstrual bleeding not associated with the treatment. During the time of the bevacizumab treatment a brightening of lesion was visible (Fig. 3B upper panel). The patient noticed a reduction of pulsation and bleeding. Pain from the lesion stopped. A follow up MR angiography with contrast enhancement detected minimal lesion growth (Fig. 3B lower panel). Genetic testing revealed a *KRAS* mosaic mutation (KRAS MIM 190070 NM_004985 c. = /183A > C p. = /(Gln61His)). The patient was therefore switched to the MEK- inhibitor trametinib to control lesion growth (Graphical Abstract). After six months of trametinib treatment a reduction in lesion size and symptom control with no pain or bleeding was achieved. The patient reported remaining pulsation of the lesion and experienced trametinib associated side effects, like significant hair loss, dry skin, and acneiform eruption of the skin.

A 66-year-old female patient with residual disease after incomplete resection of an AVM of the left thumb (Fig. 3C, upper panel) received treatment with 45 mL bevacizumab (concentration 1,5 mg/mL, in total 67,5 mg bevacizumab) by intralesional injection during digital subtraction angiography (DSA; Fig. 3C, lower left panel) to control bleeding, pulsation, and regrowth eleven months after initial surgery. Within five months of follow up the patient had no symptoms, like bleeding, pain or pulsation and the lesion is stable in size (Fig. 3C, lower right panel). Genetic testing revealed a *KRAS* mosaic mutation (KRAS c.35G > A p.Gly12Asp(G12D) in exon 2).

A 35-year-old male patient with an AVM of the upper lip (Fig. 3D, upper panel) received intralesional treatment during DSA (Fig. 3D, middle panel) with 3 mL bevacizumab (concentration 3,75 mg/mL, in total 11,25 mg bevacizumab) due to pain and pulsation of the lesion. Previous embolizations and surgeries did not achieve symptom control. Due to lesion growth after previous surgeries, no resection or biopsy for genetic testing was performed. Therefore, the mutation causing the AVM in this patient is unknown. The follow-up three months after local bevacizumab injection demonstrates a reduction of symptoms with no pain, limited pulsation, brightening and volume reduction of the lesion (Fig. 3D, lower panel).

Overall, no patient-reported side effects were observed during bevacizumab treatment. All patients noted a reduction of AVM symptoms, in one patient regression of the lesion was visible.

## Discussion

Our data show, that mechanical stress induces an upregulation of VEGF in endothelial AVM cells in vitro. VEGF, TGF-β, and their receptors are upregulated in the microenvironment of fast-flow malformations. Concordant with our findings, others demonstrated that wall shear stress on vascular smooth muscle cells exerts angiogenic effects on extracranial AVMs by increase of TGFBR1 and TGF-β1 [17, 18]. Their studies showed that biomechanical stimulation aggravates angiogenesis in AVMs. Our results show that this is attributed to the upregulation of growth factors. To target this effect therapeutically, bevacizumab and thalidomide were tested in an in vitro model for mechanical stress on CD31^+^ endothelial AVM cells. Derived from our findings bevacizumab and thalidomide mitigated VEGF induced effects in endothelial AVM cells.

VEGF-A binds to VEGFR-2 (also known as KDR or Flk-1) and induces receptor dimerization and activation, which leads to downstream signaling pathways that promote angiogenesis, endothelial cell proliferation, migration, and survival [19]. VEGF-B binds to VEGFR-1 (also known as Flt-1) and NRP-1. VEGF-B has been shown to modulate VEGFR-2 signaling indirectly by enhancing VEGF-A-induced angiogenesis and contributing to blood vessel maturation and stability [20]. It has been shown that VEGF stimulation is necessary for induction of AVM formation in the brains of adult mice [21]. Elevation of the VEGF level increases brain AVM hemorrhage and mouse mortality [22]. It is hypothesized that microvessel formation through endothelial cell migration, proliferation, and differentiation is stimulated by hypoxia, caused by incomplete resection or partial embolization of the AVM [23]. Hypoxia activates the hypoxia-inducible transcription factor (HIF-1α) signaling pathway and angiogenesis by regulating the expression of NOTCH1, angiopoietin2 (ANGPT2) and VEGF-A [24]. Our in vitro results demonstrate that the VEGF-A antibody bevacizumab inhibits angiogenic properties of endothelial AVM cells but cannot inhibit cell proliferation of AVM endothelial cells exposed to cyclic mechanical stretching.

The antiangiogenic mechanisms of action of thalidomide is the inhibition of VEGF, bFGF, and TGF-β signaling, which has been shown to contribute to the anti-inflammatory and anti-tumor effects of the drug [9, 25–27]. Thalidomide improves mural cell coverage of brain AVM vessels and reduces brain AVM hemorrhage in a mouse model [28]. Furthermore, thalidomide improves the severity and frequency of bleeding from nasal telangiectasias in HHT patients, which is likely through the promotion of vessel maturation due to an upregulation of platelet-derived growth factor-B (PDGF-B) expression [28, 29]. Thalidomide inhibits VEGF secretion, decreases the number of formed capillary tubes and increases cell adhesion to collagen in capillary formation of human endothelial cell line EA.hy 926 [30]. Our findings did not demonstrate an equally strong inhibition of AVM endothelial cell circular network formation by thalidomide compared to bevacizumab. Furthermore, HDMEC circular network formation was not impaired by thalidomide. The anti-angiogenic action of thalidomide might not solely be due to direct VEGF inhibition, but due to immunomodulatory influence on cell migration and adhesion with variable effects on different cell types [30].

A recent case report study of 18 patients with severe AVMs showed efficacious management of chronic pain, bleeding and ulceration by thalidomide therapy [23]. Bevacizumab is established as a safe and successful drug in treating epistaxis, gastrointestinal and hepatic vascular telangiectasias of HHT [7, 30, 31]. The literature on the use of bevacizumab in treating extracranial AVMs is limited. Here, we report the successful intralesional and systemic use of bevacizumab in three AVM patients with no side effects. Bevacizumab in AVM treatment reduced pain, pulsation, bleeding, and induced brightening of the lesion in all patients. In two patients bevacizumab treatment led to stable disease. The third patient showed minimal lesion growth.

The knowledge on molecular causes for AVM formation has expanded over the recent years. Somatic mutations in *KRAS, BRAF* and *MAP2K1*, which encodes the dual specificity mitogen-activated protein kinase MEK1, have been reported in sporadic AVMs [4, 32, 33]. All of the mutations lead to activation of the RAS signaling pathway inducing cell proliferation, migration and angiogenesis [23]. Trametinib, an inhibitor of MEK, has been reported for the targeted treatment of extensive AVMs [34, 35]. Therefore, genetic causes of the disease need to be targeted to reduce lesion growth, but this treatment is associated with severe side-effects [1, 34, 36–38]. We suggest that VEGF inhibitors bevacizumab and thalidomide are combined with RAS pathway inhibitors to increase treatment efficacy. By inhibiting both targets of angiogenesis simultaneously with lower drug concentrations, MEK inhibitor associated side-effects could be reduced. Furthermore, both drugs can be used solely for symptom and recurrence control after embolization and surgery.

Overall, mechanical stress increases VEGF expression in endothelial AVM cells, possibly causing the VEGF upregulation in the microenvironment of AVM cells triggering lesion growth. Therefore, we propose VEGF inhibition to control symptoms of extracranial AVM patients until genetic testing results are available for MEK inhibitor treatment or to prevent recurrence after surgery and embolization of extensive AVMs (Graphical Abstract).

## Materials and methods

### Patients

Samples of patients who had surgery on vascular malformations between 2018 and 2022 were analyzed. The study was conducted at the Department of Otorhinolaryngology, University Medical Center Regensburg, Germany, according to the principles of Helsinki and approved by the Local Ethics Committee (No. 17-854-101). All subjects, and/or their legal guardians, gave written informed consent.

### RNA isolation and PCR array

RNA was extracted from formalin-fixed and paraffin-embedded (FFPE) samples using the RNeasy FFPE Kit (Qiagen, Hilden, Germany), as described by the manufacturer. A pool of ten fast-flow malformation patients (arteriovenous malformations) was compared with a pool of ten slow-flow malformation patients (venous (n = 6) and lymphatic malformations (n = 4)). Expression of genes relevant for angiogenesis was determined with a RT2 Profiler PCR Array (PAHS-024Z, Qiagen) using RT2 SYBR Green qPCR Mastermix (Qiagen) and the LightCycler 480 Real-Time PCR System (Roche Diagnostics, Mannheim, Germany).

### Immunohistochemistry

FFPE samples (5 µm) were deparaffinized and immersed in an antigen retrieval solution (Citrate-EDTA buffer: 10 mM Citric Acid, 2 mM EDTA, 0.05% Tween-20, pH 6.2) for 20 min at 95 °C–99 °C. Sections were subsequently blocked for 30 min in TNB Blocking buffer (PerkinElmer, Boston, MA) followed by incubation with human-specific TGF-β polyclonal antibody (1:400, rabbit anti-human, Abcam, Cambridge, UK, ab92486). Immunohistochemical staining for VEGFR-2 was provided by the Department of Pathology, University Medical Center Regensburg. (Benchmark ultra, Roche, Mannheim, Germany; 1:400, rabbit anti-human, medac, Wedel, Germany, E3710). Each slide was manually scanned with a microscope at 10X, 25X or 40X magnification. We captured five areas per slide (five high-power fields, HPFs) with each showing a characteristic staining of the whole slide. Positive stained cells were counted via ImageJ (National Institutes of Health, USA) cell counter function by two independent examiners. The H-score is a method of assessing the extent of immunoreactivity, applicable to VEGF receptors. The score is obtained by the formula: 3 × percentage of strongly staining + 2 × percentage of moderately staining + percentage of weakly staining, giving a range of 0–300 [39].

### Cell isolation and culture

Single-cell suspensions were prepared from three different tissue samples of AVM patients with disease stage Schobinger III. The AVM diagnosis was confirmed by the Institute of Pathology at the University Hospital Regensburg. In all three AVM patients *KRAS* mosaic mutations were detected. Endothelial cells (EC) were selected using anti-CD31-coated magnetic beads (Miltenyi Biotec, Auburn, CA) respectively and expanded. Testing for mycoplasma contamination by qPCR was performed when cells were thawed and every 4–6 weeks thereafter. Cells were cultured on fibronectin-coated (0.1 µg/cm^2^; EMD Millipore, Billerica, MA) plates with Endothelial Cell Growth Medium-2 (EGM-2; Lonza, Allendale, NJ), which consists of Endothelial Cell Growth Basal Medium-2 (EBM-2; Lonza), SingleQuot supplements (all except hydrocortisone; Lonza), 10% heat-inactivated fetal bovine serum (FBS; Hyclone, South Logan, UT) and 1X GPS (292 mg/mL Glutamine, 100 U/milliliter (mL) penicillin, 100 mg/mL streptomycin; Mediatech Inc, Manassas, VA). Cells were cultured at 37 °C in a humidified incubator with 5% CO2.

### Cyclic mechanical stretching

To mimic the mechanical stress on high-flow malformations a mechanical stretcher was designed by the department for Science Laboratory Technology of the University Regensburg. The mechanical stretcher performs uniaxial cyclic mechanical stretch on cells in tissue culture (Supporting Fig. 1B). AVM endothelial cells (2 × 10^4^ cells/cm^2^ in 2000μL EGM-2 culture medium per well) were seeded in duplicates into 6-well plates with flexible floor membranes (BioFlex Culture Plate Untreated 6-Well, Flexcell International Corporation, Burlington, USA) for 24 h. Then, the medium was removed and either fresh EGM-2 culture medium or respective treatment (bevacizumab 1000 µg/ml or thalidomide 20 µM in EGM-2 medium) was added. The flexible membranes were stretched by a generated negative pressure of − 0,3 bar every minute. Once the threshold pressure was reached every minute pressure increased to 0 bar. The cells attached to the membrane were exposed to cyclic mechanical stretching every minute for 24 or 48 h. During the experiment the mechanical stretcher was placed at 37 °C in a humidified incubator with 5% CO_2_. A 6-well plate with flexible floor membranes placed in the incubator without stretching served as control.

### RNA isolation and quantitative reverse transcriptase PCR (qPCR)

Total cellular RNA was extracted from cultured cells with a RNeasy Micro extraction kit (Qiagen, Valencia, CA). Reverse transcriptase reactions were performed using an iScript™cDNA synthesis kit (BioRad, Hercules, CA). QPCR was performed using KAPA SYBR® FAST ABI Prism 2 × qPCR Master Mix (KAPA BioSystems, Wilmington, MA). Amplification was carried out in a StepOne™ Real-Time PCR System (Applied Biosystems, Foster City, CA). A relative standard curve for each gene amplification was generated to determine the amplification efficiency, with greater 90% considered acceptable. Fold increases in gene expression were calculated according to two delta CT method, with each amplification reaction performed in duplicates or triplicates. GAPDH was used as housekeeping gene expression reference. Primer sequences are shown in Table 1.

**Table 1:** QPCR primers

Gene	Forward	Reverse
GAPDH	GGTCGGTGTGAACGGATTTG	GTGAGCCCCAGCCTTCTCCAT
VEGFR1	GAGATGAGCTTCCTACAGCAC	TCACCGCCTCGGCTTGTCACAT

### Enzyme-linked immunosorbent assay (ELISA)

Soluble cytokine production in the supernatants of cultured AVM endothelial cells was tested by ELISA (DuoSet ELISA Development Systems; R&D Systems). Prior to use, the cell culture supernatants were centrifuged at 1200 rpm for 5 min. The DuoSet kit human VEGF (DY293B) was used according to the manufacturer’s instructions. Measurements were obtained in triplicates.

### Proliferation assay

CD31^+^ endothelial AVM cells (8 × 10^4^ cells in 3000 μL of the respective culture medium per well) were seeded in triplicates into 6-well plates. Four doses of bevacizumab and onartuzumab (250 µg/ml, 500 µg/ml, 750 µg/ml und 1000 µg/ml) and three doses of thalidomide (10 µM, 20 µM and 40 µM) in EGM-2-medium were analyzed. Concentrations were selected in accordance with current literature [12–15]. After 4 h and 24 h cells were trypsinized and counted. Proliferation was assessed by the number of cells detected after 24 h of growth.

### Angiogenesis assay

25 ng/ml VEGF-A was added to the control group and the two treatment groups (1000 µg/ml bevacizumab and 20 µM thalidomide) one hour prior to the angiogenesis assay. Wells were precoated with Matrigel (Sigma-Aldrich, St. Louis, USA) and incubated for 30 min at 37 °C. AVM endothelial cells and HDMEC, as positive control, were seeded at a density of 4 × 10^4^ cells/cm^2^ in 500 μL of EBM-2/0.1%FBS. 25 ng/ml VEGF-A and respective treatment (1000 µg/ml bevacizumab and 20 µM thalidomide) were added to the VEGF-A control and treatment conditions. After 6 h, pictures were taken with an inverted microscope (Echo Rebel Inverted Brightfield Microscope, Echo, San Diego, CA). The number of circular networks was counted per nine squares per high power field. The area of the circular networks was measured in pixels. Fiji ImageJ software (NIH) was used for analysis.

### Bevacizumab treatment of AVM patients

After interdisciplinary and multicentric discussion three patients were treated with bevacizumab. Other therapeutic options, like embolization and surgery, did not result in symptom control in all three patients. The patients were informed about the off-label use of bevacizumab in AVM treatment and possible side-effects, especially gastrointestinal, renal, and cardiovascular risks (for a detailed list of possible side-effects see AVASTIN® (bevacizumab) full prescribing information provided online by the manufacturer; https://www.gene.com/medical-professionals/medicines/avastin). The patients gave informed consent to the individual patient treatment. Before the first administration of bevacizumab blood pressure was measured, a blood test with complete blood count, a comprehensive metabolic panel, C-reactive protein test, thyroid function test, coagulation tests, B-type natriuretic peptide test, a urinalysis with urine protein test and an echocardiography was performed. Immediately before systemic bevacizumab administration, 8 mg dexamethasone were given intravenously. The day after the treatment blood and urine tests were repeated and the patient was discharged from the hospital.

A 25-year-old female patient with an AVM of the right ear and face was treated with 5 mg bevacizumab per kg/bodyweight systemically every 14 days (395 mg bevacizumab dissolved in 250 mL of 0.9% sodium chloride intravenously every 14 days) over a period of eight months. The dosage was chosen as previously described for the systemic treatment of HHT [16, 40]. A 66-year-old female patient with residual disease of an AVM of the left thumb and a 35-year-old male patient with an AVM of the upper lip received treatment with 45 mL bevacizumab (concentration 1,5 mg/mL, in total 67,5 mg bevacizumab, dissolved in 0.9% sodium chloride) and 3 mL bevacizumab (concentration 3,75 mg/mL, in total 11,25 mg bevacizumab, dissolved in 0.9% sodium chloride) by intralesional injection. The volume of bevacizumab was determined by the volume of contrast agent used to visualize the lesion during digital subtraction angiography. As the second patient showed a small lesion with low volume, a higher concentration of bevacizumab was used. The concentration was chosen as previously described for the intralesional treatment of arteriovenous malformations of the mucosa of the nose in HHT [41]. A review of the literature on intralesional use of bevacizumab in HHT showed a significant improvement of the bleeding burden (42).

### Statistics

Data was analyzed and plotted by using GraphPad Prism 9.5 (GraphPad Software). For experiments in which cells were exposed to mechanical stress or treated with drugs, the differences were assessed by one-way analysis of variance (ANOVA) followed by the post-hoc Šidák or Bonferroni test for multiple comparisons of different treatment modalities. For comparisons between fast-flow malformations and slow-flow malformations Mann Whitney test was applied. p-values ≤ 0 0.05 are considered significant. Means and standard deviations are shown in all graphs.

### Supplementary Information

Below is the link to the electronic supplementary material.

Supplementary file1 (JPG 573 KB)

Supplementary file2 (JPG 836 KB)

Supplementary file3 (DOCX 1264 KB)

## Abbreviations

ANGPT2: Angiopoietin2
AVM: Arteriovenous malformation
BRAF: B-raf protooncogene
CMS: Cyclic mechanical stretching
DSA: Digital subtraction angiography
EC: Endothelial cell
ELISA: Enzyme-linked immunosorbent assay
ERK: Extracellular signal-regulated kinases
FF: Fast-flow malformations
FGF: Fibroblast growth factors
HDMEC: Human dermal endothelial cells
HHT: Hereditary hemorrhagic telangiectasia
HIF: Hypoxia-inducible transcription factor
LM: Lymphatic malformation
MAP3K3: Mitogen-activated protein kinase kinase kinase 3
MEK: Mitogen-activated protein kinase kinase
mTOR: Mammalian target of rapamycin
NHF: Normal human fibroblasts
NOTCH1: Neurogenic locus notch homolog protein 1
NRP: Neuropilin
PCR: Polymerase chain reaction
PIK3CA: Phosphatidylinositol-4,5-bisphosphate 3-kinase catalytic subunit alpha
PDGF-B: Platelet-derived growth factor-B
RAS: Rat sarcoma
RAF: Rapidly accelerated fibrosarcoma
SF: Slow-flow malformations
TGF: Transforming growth factor
VEGF: Vascular endothelial growth factor
VEGFR: Vascular endothelial growth factor receptor
VM: Venous malformation
