# Supplementary figures and images for: Targeting the microenvironment in the treatment of arteriovenous malformations
Source: Angiogenesis. 2023 Sep 21;27(1):91–103. doi: 10.1007/s10456-023-09896-3 (PMC10881762; doi:10.1007/s10456-023-09896-3)

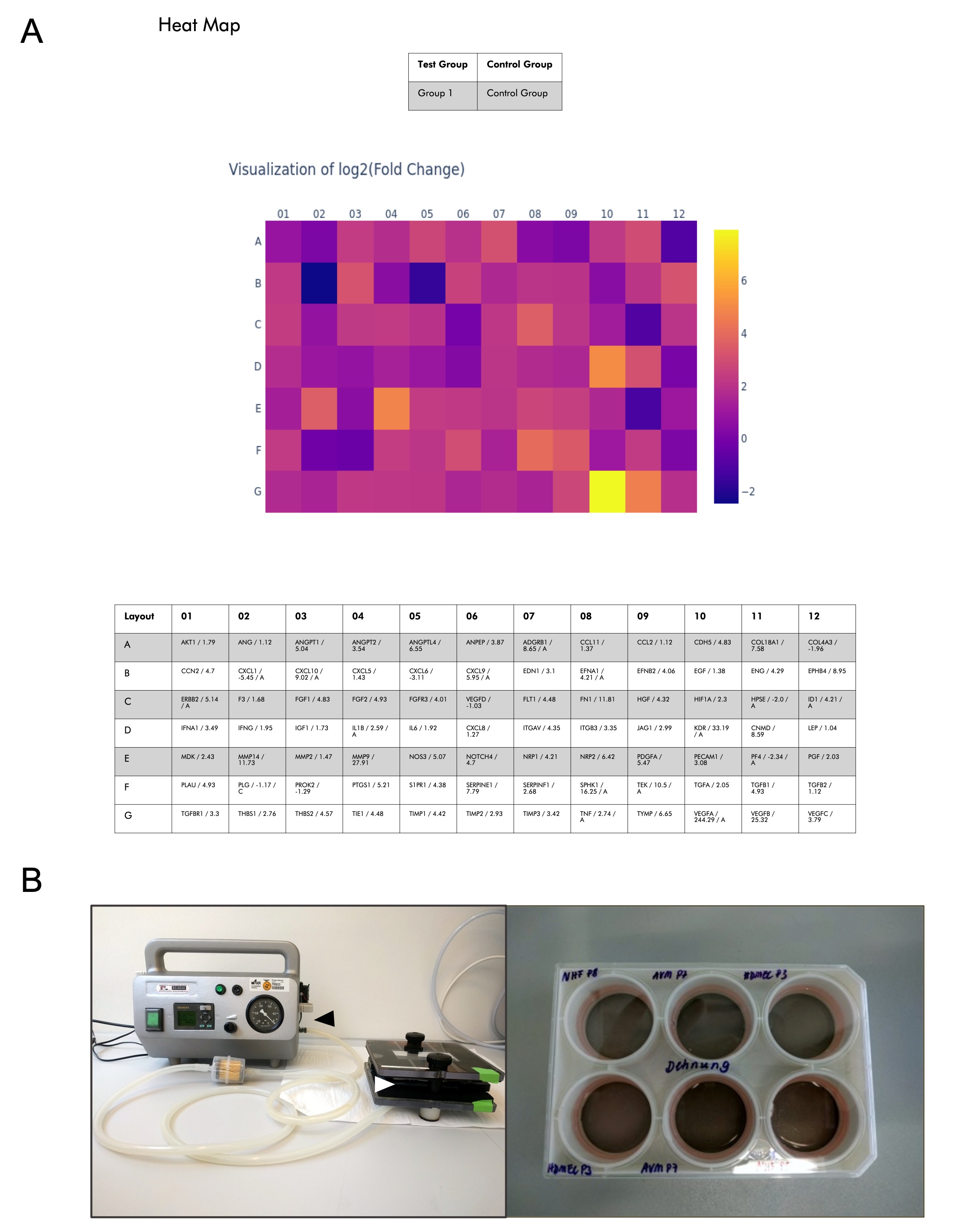

Supplement: Supplementary file 1 — Supplementary file1 (JPG 573 KB) [file 10456_2023_9896_MOESM1_ESM.jpg]

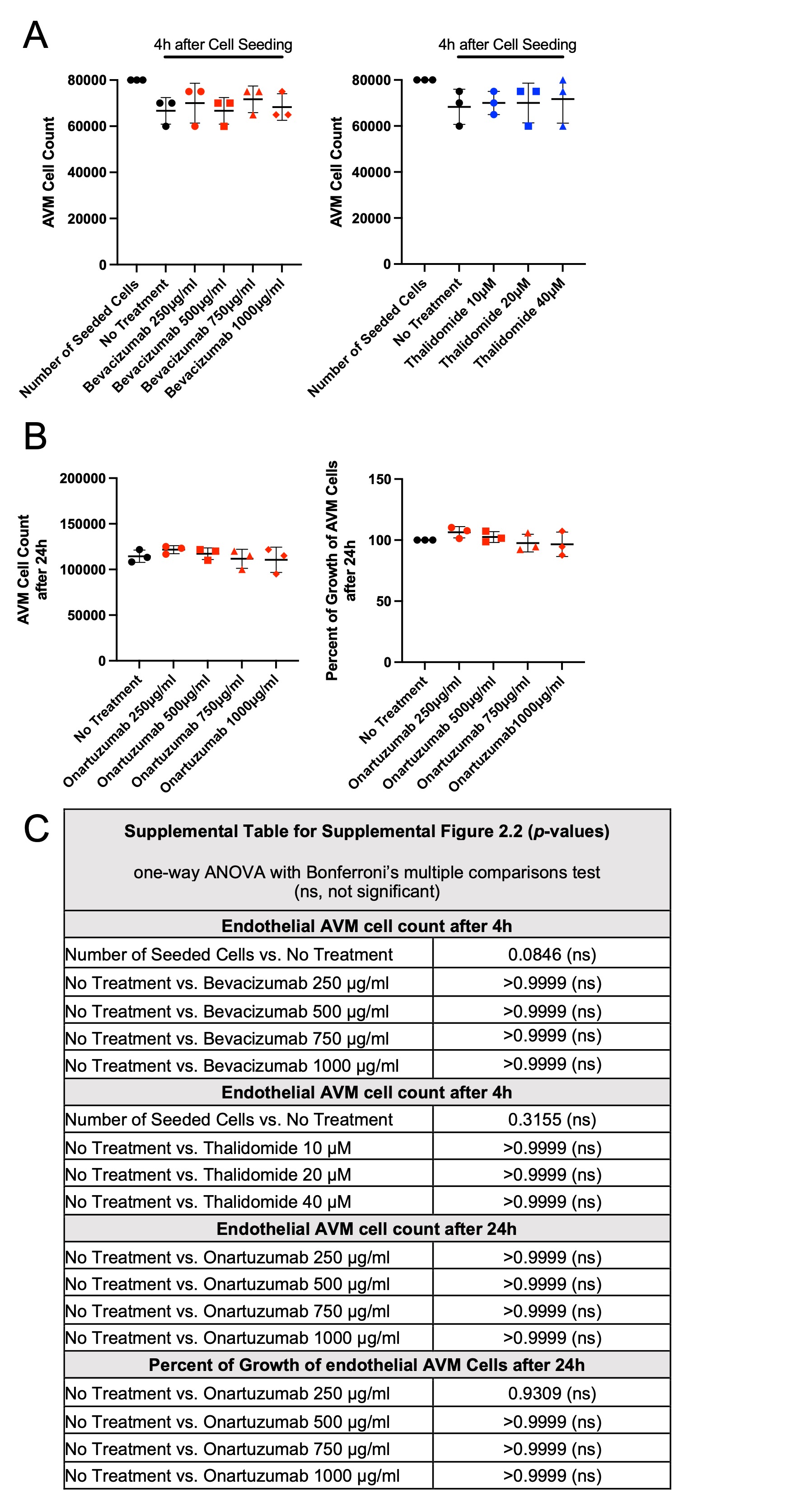

Supplement: Supplementary file 2 — Supplementary file2 (JPG 836 KB) [file 10456_2023_9896_MOESM2_ESM.jpg]
