# Supplementary material for: Targeting the microenvironment in the treatment of arteriovenous malformations
Source: Angiogenesis. 2023 Sep 21;27(1):91–103. doi: 10.1007/s10456-023-09896-3 (PMC10881762; doi:10.1007/s10456-023-09896-3)
Supplement: Supplementary file 3 — Supplementary file3 (DOCX 1264 KB) [file 10456_2023_9896_MOESM3_ESM.docx]

Supporting Figure 1. (A) Summary of PCR array results for angiogenesis (PAHS-024Z). Fold-Change (2^(- Delta Delta CT)) is the normalized gene expression (2^(- Delta CT)) in the Test Sample (fast-flow malformations) divided the normalized gene expression (2^(- Delta CT)) in the Control Sample (slow-flow malformations). (B) Display of the mechanical stretcher (left) and 6-well plate with flexible floor membrane and medium (right). Vacuum pump generating negative pressure (black triangle); stretch chamber sealed with lid to hold the 6-well plate (white triangle).

Supporting Figure 2. (A) Ascending concentrations of bevacizumab and thalidomide did not affect endothelial AVM cell attachment four hours after cell seeding. 8x10^4^ CD31^+^ endothelial AVM cells in 3000 μL of the respective culture medium per well were seeded into 6-well plates. After 4 hours of attachment cells were trypsinized and counted. (B) To exclude an unspecific IgG1 associated effect on endothelial AVM cell proliferation the monoclonal IgG1 antibody against tyrosine-protein kinase Met, onartuzumab, was tested in ascending concentrations (250 µg/ml, 500 µg/ml, 750 µg/ml und 1000 µg/m in EGM-2-medium). Treatment with onartuzumab did not impact endothelial AVM cell proliferation. This result strongly suggests a VEGF specific effect in the decrease of endothelial AVM cell proliferation due to bevacizumab treatment. (C) P-values for experiments displayed in Supporting Figure 2 A and B. P-values displayed were calculated by one-way ANOVA followed by the post hoc Bonferroni test for multiple comparisons. Means and standard deviations are shown.

| **Supplemental Table for Figure 1C (*p*-values)**  one-way ANOVA with Šídák's multiple comparisons test (ns, not significant) | |
| --- | --- |
| **VEGF mRNA levels after 24h** | |
| HDMEC-Control vs. HDMEC-CMS | 0.0260 |
| AVM-Control vs. AVM-CMS | 0.0008 |
| NHF-Control vs. NHF-CMS | 0.9909 (ns) |
| **VEGF mRNA levels after 48h** | |
| HDMEC-Control vs. HDMEC-CMS | 0.9482 (ns) |
| AVM-Control vs. AVM-CMS | 0.0002 |
| NHF-Control vs. NHF-CMS | 0.9997 (ns) |
| **VEGF protein levels after 24h** | |
| HDMEC-Control vs. HDMEC-CMS | 0.9991 (ns) |
| AVM-Control vs. AVM-CMS | 0.0044 |
| NHF-Control vs. NHF-CMS | 0.9508 (ns) |
| **VEGF protein levels after 48h** | |
| HDMEC-Control vs. HDMEC-CMS | 0.9990 (ns) |
| AVM-Control vs. AVM-CMS | 0.0019 |
| NHF-Control vs. NHF-CMS | 0.9991 (ns) |

| **Supplemental Table for Figure 2A (*p*-values)**  one-way ANOVA with Bonferroni’s multiple comparisons test (ns, not significant) | |
| --- | --- |
| **Endothelial AVM cell count after 24h** | |
| No Treatment vs. Bevacizumab 250 µg/ml | >0.9999 (ns) |
| No Treatment vs. Bevacizumab 500 µg/ml | 0.2979 (ns) |
| No Treatment vs. Bevacizumab 750 µg/ml | 0.1161 (ns) |
| No Treatment vs. Bevacizumab 1000 µg/ml | 0.0176 |
| **Endothelial AVM cell count after 24h** | |
| No Treatment vs. Thalidomide 10 µM | 0.6916 (ns) |
| No Treatment vs. Thalidomide 20 µM | 0.1487 (ns) |
| No Treatment vs. Thalidomide 40 µM | 0.0104 |
| **Percent of growth of endothelial AVM cells after 24h** | |
| No Treatment vs. Bevacizumab 250 µg/ml | 0.5462 (ns) |
| No Treatment vs. Bevacizumab 500 µg/ml | 0.0202 |
| No Treatment vs. Bevacizumab 750 µg/ml | 0.0045 |
| No Treatment vs. Bevacizumab 1000 µg/ml | 0.0002 |
| **Percent of growth of endothelial AVM cells after 24h** | |
| No Treatment vs. Thalidomide 10 µM | 0.2873 (ns) |
| No Treatment vs. Thalidomide 20 µM | 0.0336 |
| No Treatment vs. Thalidomide 40 µM | 0.0011 |

| **Supplemental Table for Figure 2B (*p*-values)**  one-way ANOVA with Bonferroni’s multiple comparisons test (ns, not significant) | |
| --- | --- |
| **Area of circular networks (endothelial AVM cells)** | |
| VEGF-A vs. No Treatment | 0.0121 |
| VEGF-A vs. + Bevacizumab | 0.0056 |
| VEGF-A vs. + Thalidomide | 0.0613 (ns) |
| **Number of circular networks (endothelial AVM cells)** | |
| VEGF-A vs. No Treatment | 0.0216 |
| VEGF-A vs. + Bevacizumab | 0.0047 |
| VEGF-A vs. + Thalidomide | 0.0595 (ns) |
| **Area of circular networks (HDMEC)** | |
| VEGF-A vs. No Treatment | 0.0014 |
| VEGF-A vs. + Bevacizumab | 0.0594 (ns) |
| VEGF-A vs. + Thalidomide | 0.2673 (ns) |
| **Number of circular networks (HDMEC)** | |
| VEGF-A vs. No Treatment | 0.0153 |
| VEGF-A vs. + Bevacizumab | 0.2969 (ns) |
| VEGF-A vs. + Thalidomide | 0.6211(ns) |

| **Supplemental Table for Figure 2C (*p*-values)**  one-way ANOVA with Bonferroni’s multiple comparisons test (ns, not significant) | |
| --- | --- |
| **VEGF mRNA levels after 24h** | |
| No Treatment vs. Bevacizumab 1000 µg/ml | 0.2894 (ns) |
| No Treatment vs. Thalidomide 20 µM | >0.9999 (ns) |
| CMS - No Treatment vs. CMS - Bevacizumab 1000 µg/ml | 0.1573 (ns) |
| CMS - No Treatment vs. CMS - Thalidomide 20 µM | 0.0266 |
| **VEGF protein levels after 24h** | |
| No Treatment vs. Bevacizumab 1000 µg/ml | 0.0106 |
| No Treatment vs. Thalidomide 20 µM | 0.1683 (ns) |
| CMS - No Treatment vs. CMS - Bevacizumab 1000 µg/ml | 0.0007 |
| CMS - No Treatment vs. CMS - Thalidomide 20 µM | >0.9999 (ns) |
| **AVM cell count after 24h** | |
| No Treatment vs. Bevacizumab 1000 µg/ml | 0.0263 |
| No Treatment vs. Thalidomide 20 µM | 0.0060 |
| CMS - No Treatment vs. CMS - Bevacizumab 1000 µg/ml | >0.9999 (ns) |
| CMS - No Treatment vs. CMS - Thalidomide 20 µM | >0.9999 (ns) |
